# The effect of climate on melioidosis incidence in Townsville, Australia: a dry tropical region

**DOI:** 10.1265/ehpm.22-00177

**Published:** 2023-06-07

**Authors:** Vibooshini Ganeshalingam, Mirjam Kaestli, Robert E Norton, Ian Gassiep

**Affiliations:** 1 Faculty of Medicine, University of Queensland, Brisbane, Queensland, Australia; 2 Department of Medicine, Townsville University Hospital; 3 Department of Infectious Diseases, Mater Hospital Brisbane, South Brisbane, Queensland, 4101, Australia; 4 Department of Medicine, Princess Alexandra Hospital, Woolloongabba, Queensland, 4102; 5 Pathology Queensland, Royal Brisbane & Women’s Hospital, Herston, Queensland, 4029, Australia; 6 Pathology Queensland, Townsville University Hospital, Townsville, Queensland, 4814, Australia; 7 University of Queensland Centre for Clinical Research, Royal Brisbane and Woman’s Hospital, Herston, Queensland, 4029, Australia; 8 Menzies School of Health Research, Charles Darwin University, Darwin, Northern Territory, Australia; 9 Faculty of Medicine, University of Queensland, St.Lucia, Brisbane, Queensland, Australia

**Keywords:** Melioidosis, Weather, Rainfall, Humidity, Cloud cover, Northern Australia, Townsville

## Abstract

**Background:**

Townsville is in the dry tropics in Northern Australia and an endemic region for melioidosis. Melioidosis is an infectious disease caused by *Burkholderia pseudomallei*, a soil dwelling organism. The incidence of melioidosis is associated with high levels of rainfall and has been linked to multiple weather variables in other melioidosis endemic regions such as in Darwin. In contrast to Townsville, Darwin is in the wet-dry tropics in Northern Australia and receives 40% more rainfall. We assessed the relationship between melioidosis incidence and weather conditions in Townsville and compared the patterns to the findings in Darwin and other melioidosis endemic regions.

**Method:**

Performing a time series analysis from 1996 to 2020, we applied a negative binomial regression model to evaluate the link between the incidence of melioidosis in Townsville and various weather variables. Akaike’s information criterion was used to assess the most parsimonious model with best predictive performance. Fourier terms and lagged deviance residuals were included to control long term seasonal trends and temporal autocorrelation.

**Results:**

Humidity is the strongest predictor for melioidosis incidence in Townsville. Furthermore, the incidence of melioidosis showed a three-times rise in the Townsville region when >200 mm of rain fell within the fortnight. Prolonged rainfall had more impact than a heavy downpour on the overall melioidosis incident rate. There was no statistically significant increase in incidence with cloud cover in the multivariable model.

**Conclusion:**

Consistent with other reports, melioidosis incidence can be attributed to humidity and rainfall in Townsville. In contrast to Darwin, there was no strong link between melioidosis cases and cloud cover and nor single large rainfall events.

## Introduction

The causative agent of melioidosis, *Burkholderia pseudomallei*, a Tier 1 select agent, is endemic in Southeast Asia and Northern Australia with increased incidence associated with high levels of rainfall [1]. Melioidosis patients commonly present acutely unwell with pneumonia and septicaemia. However, the disease presentation may be indolent such as osteomyelitis. In addition, reactivation of latent infection may occur many decades after exposure [2].

The annual incidence of infection in an endemic area may be affected by a number of weather or climate related variables including humidity, rainfall, and severe weather events such as monsoons and tropical cyclones [1, 3–6]. Rainfall is thought to increase bacterial concentration in topsoil and the rhizosphere via the rise in the water table [8]. Severe weather events and wind are associated with transmission of bacteria-contaminated aerosols resulting in acquisition of infection via inhalation [6–11]. A Taiwan study demonstrated that wind direction and speed contributed to aerosolization of Burkholderia pseudomallei [6]. In Cambodia, there was a non-significant association between lung infection and wind speed [8]. Additionally, previous data reveal that heavy rainfall 14-days prior to hospitalisation is an independent risk factor for more severe disease, suggesting that there is a greater organism burden at the time of acquisition [3].

A time-series analysis performed over a 23-year period in Darwin, Australia demonstrated the association of the melioidosis incidence rate with a number of meteorological variables [1]. Aside from rainfall, cloud cover, dewpoint, and maximum temperature were all associated with an increased risk of melioidosis acquisition. This analysis also demonstrated an association between a 14-day lag of rainfall events and case incidence, which is in keeping with the incubation period of *B. pseudomallei* [12].

In contrast to Darwin, the city of Townsville in North Queensland, Australia is a dry tropical region. In Townsville the wet season extends from November to April, however the average yearly rainfall is substantially lower. The mean annual rainfall in Darwin from 1991–2020 was 1,832 mm compared to 1,095 mm in Townsville over the same time period [13]. In Darwin, the peak rainfall month is January, with an average of 470 mm. Whereas Townsville’s peak rainfall month is February, with an average of 338 mm.

Due to these regional differences the authors aimed to assess the meteorological factors associated with melioidosis incidence in a dry tropical region as compared to a wet-dry tropical region. The six meteorological variables that were chosen for this study were previously linked with increased incidence of melioidosis cases [1, 3, 6, 8]. They are rainfall, humidity, cloud cover, dew point, wind speed and minimum/maximum temperature. We analysed the volume of rainfall and compared the incidence of melioidosis with specified time delay from rainfall to melioidosis presentation. We compared the findings of our study to the Darwin study [1, 3].

## Methods

Townsville is a city in North Queensland with a local population of approximately 195,000 inhabitants and is serviced by Pathology Queensland Townsville laboratory, the state melioidosis reference centre [14]. All culture-confirmed melioidosis cases identified between January 1, 1996 and December 31, 2020 were included in the analysis. The state-wide electronic database was used to access each patient’s residential location. Only patients residing within the Townsville region were included [14]. Weather data were obtained from the Australian Bureau of Meteorology, using the Townsville airport weather station data [15].

### Exploratory analysis

The weather variables chosen for initial analysis included rainfall (mm), relative humidity at 3pm (%), cloud cover (8^th^), dewpoint (°C), wind speed (km/h), and minimum/maximum temperature (°C). The data was binned into 7 or 14 day-bins. This allowed the minimisation of zeroes and also increased the data information with cross correlation. Lags between 0 to 2 for weather variables were assessed. Patterns between weather predictors and cases were inspected with scatter plots, cross-correlation analyses and time series plots.

### Statistical analysis

Data were analysed using STATA version 16 statistical software package (StataCorp, College Station, Texas). In order to model the association between the melioidosis incidence rate and six weather variables, a negative binomial regression model was used to account for over-dispersion of the data. Both uni- and multivariable analyses were performed. The Akaike’s information criterion (AIC) was used to compare the predictive performance of the six weather variables at different time lags for the melioidosis incidence rates in univariate models. For the multivariable analysis, variables with a p-value ≤0.1 in the univariate model were included, and the AIC was used to assess the most parsimonious multivariable model with best predictive performance. Fourteen-day binned data were used based on strongest cross-correlation patterns. Fourier terms were included in order to avoid potential spurious associations between the melioidosis incidence rate and weather variables due to their shared strong seasonal patterns. Deviance residuals of lag 1 and 2 were added to address temporal autocorrelation [16]. An offset variable for population (natural log of the population) was added to obtain incidence rates and account for population growth during the time period of the study. Model residuals were inspected for lack of patterns across predictors and time, and the variance inflation factor of predictor variables calculated to ensure lack of collinearity. Multiplicative interactions between weather variables were also explored but did not improve the model fit.

## Results

**Fig. 1:**
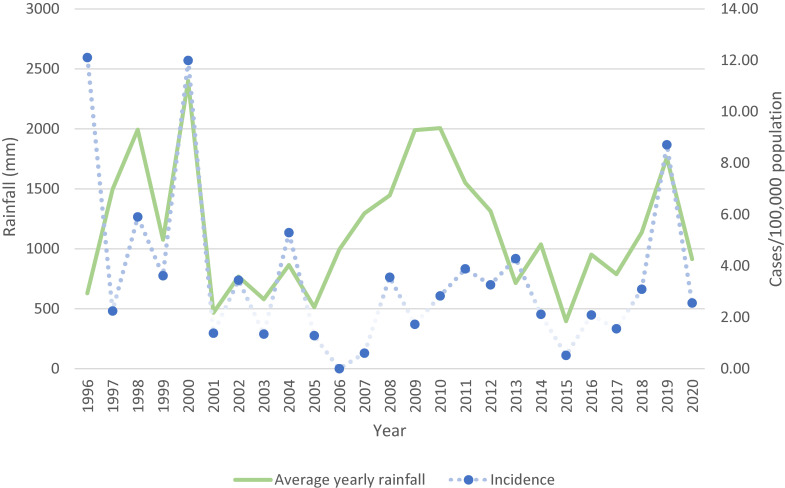
Incidence of melioidosis cases in relation to total annual rainfall in Townsville, Australia

During the study period 228 cases of culture-confirmed melioidosis in patients residing in the Townsville region were identified. The average incidence for the total population was 3.6 cases/100,000 population. Figure 1 shows the association between total annual rainfall and melioidosis incidence. Both 2000 and 2019 experienced a spike in total rainfall and melioidosis cases with an incidence of 12 and 8.7 cases/100,000 respectively. Eleven cases each were identified within 30-days following tropical cyclone Tessi in April 2000 and a major flood event in January 2019.

**Fig. 2:**
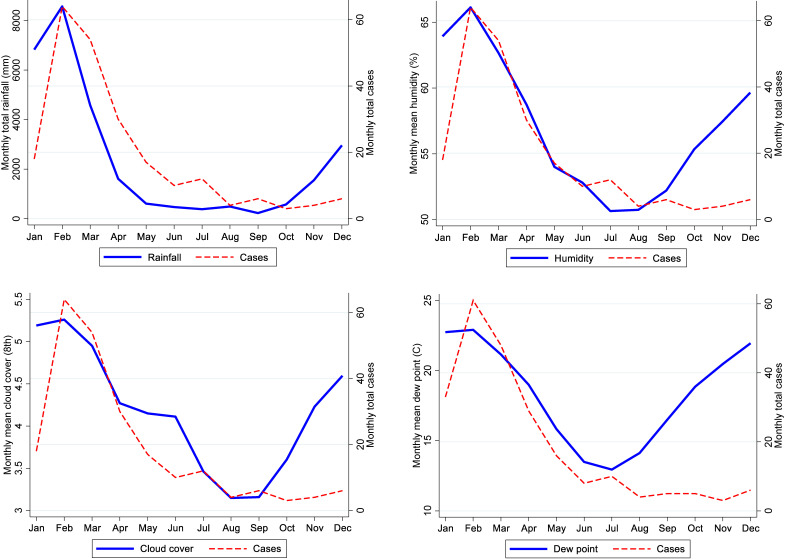
Monthly melioidosis cases associated with rainfall, humidity, cloud cover, and dew point

The greatest proportion of monthly cases were identified in February and March with a total of 63 (28%) and 54 (24%) cases respectively. These values correspond with the highest total rainfall months of January and February (Fig. 2). Additionally, both high humidity and cloud cover demonstrated a similar trend and were highest during that time. The total number of cases occurring during the wet season was 176 (77%).

Cross-correlation analysis of melioidosis cases and weather variables showed strongest correlations with 0, 1, or 2-lags of rainfall using 14-day binned data. The meteorological variables with the greatest association to melioidosis incidence were humidity, rainfall, cloud cover, and dewpoint (Table 1, Fig. 2). Due to collinearity of humidity with dewpoint, the latter was removed from the final model. As shown in Table 1A, humidity in the current fortnight showed the strongest association with melioidosis incidence rate in a univariate model followed by total rainfall in the same period. Prolonged rainfall within a 2-week period was associated with the highest incidence rate ratio (IRR). A total volume exceeding 300 mm was associated with an IRR: 3.45 (95% CI 1.89–6.27, p: 0.001). Weather events which occurred within the same 2-week period were better predictors for an increase in melioidosis infections compared to events occurring in either the previous 2- or 4-week period.

**Table 1A:** Weather variables with 0–2 lags predicting the incidence rate ratio of melioidosis

**Explanatory variables**	**Unit**	**Lag**	**Delta AIC**	**IRR**	**95% CI**	**P Value**
Humidity	%	No lag	0.0	1.06	1.03–1.08	<0.001***
Rainfall	mm	No lag	2.7	1.002	1.001–1.003	<0.001***
Rain >200 mm	Yes/No	No lag	4.6	3.08	1.85–5.14	<0.001***
Rain >100 mm	Yes/No	No lag	4.7	2.58	1.67–3.98	<0.001***
Rain >300 mm	Yes/No	No lag	7.1	3.45	1.89–6.27	<0.001***
Humidity	%	1	8.8	1.04	1.02–1.07	<0.001***
Rainfall	mm	1	8.8	1.002	1.000–1.003	0.001**
Rain >60 mm 24 h	Yes/No	No lag	9.8	2.12	1.39–3.23	<0.001***
Cloud cover	1/8	No lag	9.9	1.30	1.12–1.51	0.001**
Cloud cover	1/8	1	10.0	1.32	1.13–1.53	0.001**
Rain >200 mm	Yes/No	1	10.1	2.37	1.38–4.05	0.002**
Rain >80 mm 24 h	Yes/No	No lag	10.9	2.05	1.35–3.12	0.001**
Rain >100 mm	Yes/No	1	11.5	1.96	1.27–3.03	0.002**
Rain >40 mm 24 h	Yes/No	No lag	11.7	1.95	1.30–2.92	0.001**
Rain >80 mm 24 h	Yes/No	1	12.6	1.81	1.19–2.73	0.005**
Rain >60 mm 24 h	Yes/No	1	13.1	1.78	1.18–2.68	0.006**
Rain >300 mm	Yes/No	1	13.5	2.40	1.25–4.60	0.008**
Rain >40 mm 24 h	Yes/No	1	14.9	1.63	1.10–2.41	0.014*
Cloud cover	1/8	2	16.8	1.17	0.99–1.37	0.057
Rain >200 mm	Yes/No	2	19.9	0.92	0.51–1.64	0.779
Rain >300 mm	Yes/No	2	19.9	1.09	0.55–2.13	0.793
Rainfall	mm	2	20.1	0.999	0.998–1.001	0.919
Rain >40 mm 24 h	Yes/No	2	20.2	1.12	0.76–1.67	0.547
Rain >80 mm 24 h	Yes/No	2	20.2	1.08	0.71–1.63	0.712
Humidity	%	2	20.3	1.01	0.98–1.03	0.638
Rain >60 mm 24 h	Yes/No	2	20.4	1.01	0.67–1.52	0.938
Rain >100 mm	Yes/No	2	20.4	1.04	0.66–1.63	0.843

Negative binomial regression model to predict the incidence rate ratio (IRR) of melioidosis cases in Townsville, Australia based on 14-day binned data and weather variables as predictors. Each row represents a different model. All models accounted for population growth, seasonal bias and temporal autocorrelation (see methods). “Rain >200 mm” refers to total rainfall above 200 mm in the 2-weekly bin while “Rain >60 mm 24 h” refers to the occurrence of a rainfall event with more than 60 mm within 24 hours in the 2-weekly bin.

In the multivariable model, the incidence of melioidosis increased by an average of 1% (95% Cl: 1.00–1.03, p = 0.022), and 2% (95% CI: 1.01–1.03, p < 0.001) for each additional 10 mm of rainfall in the current and previous fortnight while accounting for humidity and cloud cover. A 10% increase in humidity within the same fortnight increased the case incidence rate by 1.3-fold on average (p = 0.040) while accounting for rainfall and cloud cover.

Cloud cover increased the case incidence by 1.3-fold in both the same and previous fortnight in the univariate model (p = 0.001 for both). However once accounting for rainfall and humidity in the multivariable model, cloud cover was no longer associated with the melioidosis incidence rate (p = 0.138).

## Discussion

*B. pseudomallei* is a soil and water dwelling environmental organism [10, 17]. Multiple studies have demonstrated the association between extreme weather events and increased incidence of melioidosis [7, 9]. While climatic factors associated with melioidosis have been well described in wet tropical regions, there are limited data regarding the dry tropics [1, 6, 13]. The study in Darwin showed that heavy rainfall was associated with an increased incidence of melioidosis. Our data showed that in Townsville the total rainfall over a previous fortnight was more of a factor in the incidence of melioidosis infection. Of all the variables assessed, humidity was strongly associated with increased incidence in this study.

There are multiple meteorological features that are likely to contribute to a difference in melioidosis incidence between regions. These include the total annual rainfall, days with rainfall, and number of cloudy days in Townsville being on average 40% lower than that seen in Darwin. The relative mean humidity however was similar [13]. In Darwin, >300 mm of rainfall in a 14-day period was associated with a 1.6 times increase in melioidosis incidence while our data demonstrated 3.5 times increase for that amount of rainfall [1]. Cloud cover in the preceding 14 days was associated with a 29% increase in incidence in Darwin, while it was not significant in our multivariable model. The reason for this difference is unclear but may reflect prolonged low volume rainfall in Townsville as opposed to more frequent heavy downpours in Darwin. This theory is further supported by the total rainfall over a fortnight having a larger impact on the melioidosis incidence in Townsville compared to heavy downpours.

Both humidity and dewpoint have been recognised as factors associated with melioidosis incidence [1, 18]. These variables are not included together in prior models nor in this analysis due to high correlation leading to collinearity issues. In this study high humidity was strongly linked to melioidosis cases. A Singaporean study only demonstrated an association between a 2-week lagged humidity and case incidence. Our data demonstrate a statistically significant association with 0 and 1 lag humidity (Table 1A). Additionally, the greatest incidence rate ratio was found with 14-day binned humidity data and no lags. This may in part be due to a more consistent mean humidity throughout the year in Singapore compared with Townsville.

**Fig. 3:**
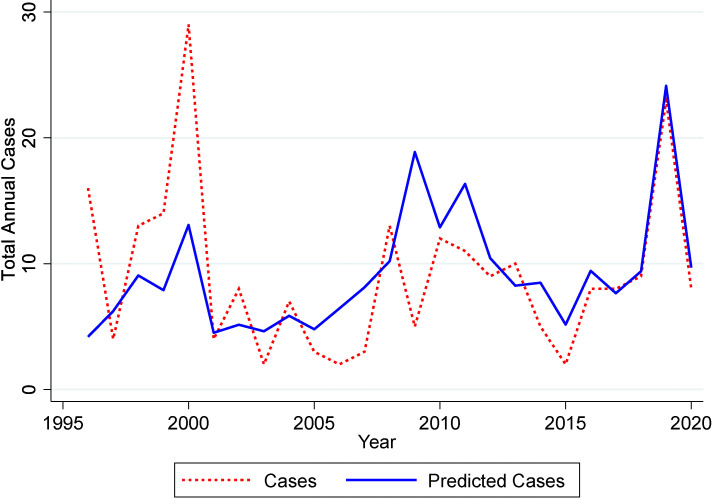
Observed and predicted melioidosis cases The predicted cases are based on the multivariable model presented in Table 1B.

The multivariable model was unable to predict the high case numbers in 1996 (Fig. 3). It is unclear why this year had so many cases as the annual total rainfall was comparatively low and there were no cyclone or flood events. Additionally, the model was not able to account for the surge in cases associated with the cyclone in 2000. Construction and soil disturbances have been suggested as potential associated factors in disease incidence. However, in Townsville during this period, there was no significant soil disturbances to account for the number of cases reported. Despite this, our model was surprisingly accurate at predicting the total cases associated with the major flood that occurred in 2019. In 2009 ex-tropical cyclone Charlotte resulted in above average rainfall, however the predominant feature was that of tidal destruction and not suburban flooding with less cases observed than the model predicted.

**Table 1B:** Multivariable model with set of weather variables which best predicted the melioidosis incidence rate

**Variable**	**Lag**	**IRR**	**95% CI**	**p-value**
Rainfall (mm)	No lag	1.001	1.000–1.003	0.022
Rainfall (mm)	1	1.002	1.001–1.003	<0.001
Humidity (%)	No lag	1.034	1.001–1.067	0.040
Cloud cover (1/8^th^)	2	1.125	0.963–1.316	0.138
*cos_1*	*No lag*	*0.445*	*0.327–0.607*	*<0.001*
*sin_1*	*No lag*	*1.175*	*0.826–1.671*	*0.371*
*sin_2*	*No lag*	*0.725*	*0.546–0.962*	*0.026*
*Deviance residual*	*1*	*1.094*	*0.871–1.375*	*0.440*
*Deviance residual*	*2*	*1.269*	*1.014–1.588*	*0.038*

Multivariable negative binomial regression model to predict the incidence rate ratio (IRR) of melioidosis cases in Townsville, Australia based on 14-day binned data and weather variables as predictors. The model accounted for population growth, seasonal bias and temporal autocorrelation (see methods) and was chosen based on the lowest AIC amongst multivariable models tested.

There are a few limitations of this study. The primary one was not assessing the association between groundwater levels and melioidosis incidence. This was due to the authors being unable to obtain groundwater data for the study period. This is a notable exclusion as previous work has suggested the movement of *B. pseudomallei* to the soil surface with an increase in groundwater levels [19]. The fluctuation of groundwater levels has also been linked to melioidosis incidence [1]. Additional work demonstrated recovery of *B. pseudomallei* from groundwater seeps and therefore the potential to distribute the organism downstream [20, 21]. These studies clearly show the association between groundwater levels, superficial groundwater seeps, and a *B. pseudomallei* contaminated environment resulting in increased melioidosis incidence. Another limitation is that rainfall data from a single station in Townsville (Townsville aero) was included. This station is located 2 kilometres from Townsville city where most of the cases are from. However, it may not accurately reflect the rainfall for cases in surrounding suburbs. An additional limitation of this study was that not all clinical data was available for all patients. We were unable to distinguish between specific clinical presentations and rainfall. Though majority of cases presented acutely, there was a small number of indolent melioidosis presentation. It is not possible to link the indolent melioidosis presentation to weather events. A further limitation was that though no culture positive melioidosis cases were missed, there might have been small number of undiagnosed melioidosis cases or those who presented to other region hospital that was missed.

Despite these limitations, this study illustrates the similarities and highlights the differences of the impact of weather elements on the melioidosis incidence between various regions of Australasia.

## Conclusion

This study has demonstrated significant difference in incidence of melioidosis associated with different weather patterns between two regions of melioidosis endemicity in Australia. The total annual rainfall, days with rainfall and number of cloudy days, in the dry tropics of Townsville is on average lower than that seen in the wet tropics of Darwin. Our results reveal a stronger association with periods of prolonged rainfall in the Townsville region, as compared to heavy downpour events in a short period of time. This contrasts with Darwin where the latter was strongly linked to a rise in melioidosis cases. Furthermore, this study demonstrates the complex interplay between disease and weather events. There will clearly be factors other than weather related variables which will play a part in the emergence of melioidosis in vulnerable populations. The findings of this study can contribute to educating the community on prevention of melioidosis during severe weather events such as to avoid flood waters, staying indoors during a severe weather event, to be more vigilant in screening for melioidosis amongst health professionals within the fortnight post these events. This data may be used from a public health perspective to aid in regional preventative measures and for resource allocation to coincide with these weather events.

## Declaration

### Availability of data and materials

Data available on request from authors.
